# Characterization of the Metallothionein Gene Family in *Avena sativa* L. and the Gene Expression during Seed Germination and Heavy Metal Stress

**DOI:** 10.3390/antiox12101865

**Published:** 2023-10-15

**Authors:** Wiktoria Konieczna, Agnieszka Mierek-Adamska, Natalia Chojnacka, Marcel Antoszewski, Aleksandra Szydłowska-Czerniak, Grażyna B. Dąbrowska

**Affiliations:** 1 Department of Genetics, Faculty of Biological and Veterinary Sciences, Nicolaus Copernicus University in Toruń, Lwowska 1, 87-100 Toruń, Poland; wpyrkosz@doktorant.umk.pl (W.K.); mant@doktorant.umk.pl (M.A.); 2 Centre for Modern Interdisciplinary Technologies, Nicolaus Copernicus University in Toruń, Wileńska 4, 87-100 Toruń, Poland; 3 Department of Analytical Chemistry and Applied Spectroscopy, Faculty of Chemistry, Nicolaus Copernicus University in Toruń, Gagarina 7, 87-100 Toruń, Poland; olasz@umk.pl

**Keywords:** oat, metallothioneins, promoter, germination, zinc, cadmium, antioxidants

## Abstract

Metallothioneins (MTs) are a family of small proteins rich in cysteine residues. The sulfhydryl group of metallothioneins can bind to metal ions, maintaining metal homeostasis and protecting the cells from damage caused by toxic heavy metals. Moreover, MTs can function as reactive oxygen species scavengers since cysteine thiols undergo reversible and irreversible oxidation. Here, we identified 21 metallothionein genes (*AsMT*s) in the oat (*Avena sativa* L.) genome, which were divided into four types depending on the amino acid sequences of putative proteins encoded by identified genes. Analysis of promoter sequences showed that *MTs* might respond to a variety of stimuli, including biotic and abiotic stresses and phytohormones. The results of qRT-PCR showed that all four types of *AsMT*s are differentially expressed during the first 48 hours of seed germination. Moreover, stress induced by the application of zinc, cadmium, and a mixture of zinc and cadmium affects the expression of oat *MTs* variously depending on the MT type, indicating that *AsMT1-4* fulfil different roles in plant cells.

## 1. Introduction

Metallothioneins (MTs) are low-molecular-weight, cysteine-rich proteins present in microorganisms, animals, and plants [1,2,3,4]. Upon the discovery of metallothioneins, they were described as proteins that bind to cadmium ions [5], and further, it was shown that MTs can bind to a variety of heavy metal ions including zinc and copper [4]. The first plant metallothionein (pMT) was discovered in wheat embryos and was named an early cysteine-labelled protein (E_c_ protein) [6]. Its mRNA was present in dry wheat embryos but not in germinated embryos [7].

Cysteine (Cys, C) is a unique amino acid thanks to the presence of a thiol group. The sulfhydryl group (-SH) is the high-affinity binding site of several metals including zinc and cadmium [8]. MTs are found throughout all kingdoms of living organisms and are highly diversified. The most common feature of all MTs is the high content of cysteines, even up to 30% of all amino acids are cysteines [9]. Plant metallothioneins are more diversified than MTs from other groups of organisms, and they are divided into four types (MT1—MT4) based on the arrangement and number of Cys residues [10]. Type-1 pMTs have 12 Cys residues, type-2 pMTs have 14 Cys residues, type-3 pMTs have 10 Cys residues, and type-4 pMTs have 17 Cys residues. The Cys residues in type-1–3 pMTs are grouped into two domains, whereas the Cys of type-4 pMTs are grouped into three domains [11,12,13]. In addition, some plant MTs have histidine (His, H) residues, which is not a common feature of MTs. It has been shown that those histidines participate in metal binding [14]. Histidines are also present in some bacterial MTs, e.g., His in bacterial SmtA from *Synechococcus* PCC7942 stabilize the protein folding and impact metal cluster charge [15]. Moreover, according to Pearson’s theory of hard and soft acids and bases (HSAB theory) [16], His could allow metallothioneins to differentiate between structurally similar zinc and cadmium ions; i.e., MTs fold properly only in the presence of zinc but not in the presence of cadmium [17].

Cysteine thiols can undergo reversible and irreversible oxidation [8]. Thus, MTs can act as reactive oxygen species (ROS) scavengers [18]. At low concentrations, ROS are important signaling molecules. However, in excess, ROS cause extensive damage to proteins, DNA, and lipids, disturbing cellular functioning [19]. Plants have developed diversified mechanisms that allow them to maintain redox homeostasis. An enzymatic antioxidant system consists of enzymes like superoxide dismutase (SOD), peroxidase (PX), and catalase (CAT). Moreover, non-enzymatic compounds like ascorbic acid, reduced glutathione, phenolics, and proline serve as antioxidants [20,21,22,23]. Numerous lines of evidence show that pMTs could be a part of the plant non-enzymatic antioxidant system. We showed previously that *Brassica napus* L. MTs can diminish ROS damage when overexpressed in *E. coli* cells [24]. Subsequently, we found that the expression of oat *MTs* correlates positively with the increased level of antioxidant enzymes like SOD, CAT, and PX [25]. Moreover, transgenic *Arabidopsis thaliana* (L.) Heynh. overexpressing date palm *MT2* had improved ROS scavenging ability [26]. MT3a from *Gossypium hirsutum* L. was shown to scavenge superoxide and hydroxyl radical in vitro [27]. Plant *MT* expression can be induced by various stress conditions, including cold [28], drought [23,25], and biotic stress [29]. Furthermore, plants that are overexpressing MTs exhibit higher tolerance to cadmium [30,31], drought [32], freezing, and salt stress [33]. Several studies have shown that the expression of *pMTs* also changes during plant development [34,35], including seed germination [34,36,37]. These observations indicate that MTs are crucial in plants’ development, growth, and survival in adverse environmental conditions.

Metallothionein gene families have been investigated in numerous plants such as *A. thaliana* [38], *Oryza sativa* L. [39,40], *Zea mays* L. [41] *Nicotiana tabacum* L. [13], and *Cucumis sativus* L. [13]. However, relatively little is known about the diversity of *MT* genes in polyploid plants, e.g., *B. napus* [37,42]. Oat (*Avena sativa* L.) is a cereal crop from the family *Poaceae*, widely known for its healthy and nutritious properties. The seeds of modern oat cultivars are rich in minerals, including Zn, Cu, Ca, Fe, and Mg [43]. Moreover, oat seeds have a high content of proteins [44], antioxidants [45], vitamins E and B [46], and β-glucan. In particular, oat β-glucan has been afforded more attention recently, thanks to its cholesterol-lowering properties [47]. Oat consumption has many positive health effects since it can help reduce hyperglycemia, hyperinsulinemia, hypercholesterolemia, and hypertension. It is recommended to eat oats to prevent cardiovascular diseases [48]. The genus *Avena* consists of diploid (AA or CC genomes), tetraploid (AABB, AACC, CCCC, or CCDD genomes), and hexaploid species (AACCDD genomes). It is believed that the hexaploid oat (AACCDD, *2n = 6x =* 42; ~12,500 Mb) arose from hybridization between a CCDD allotetraploid and an AA diploid [49,50]. The sequencing of the oat genome was difficult due to the big size of its genome (12.5 Gb) and the mosaic structure of the chromosomes [51]. In 2019, the first chromosome-scale assemblies of oat diploid species were published [52]; the first hexaploid oat reference genome was published in 2021 [53]; and in 2022, the *A. sativa* cv. Sang genome sequence was published [51]. With the oat genome sequence available, it is expected that the amount of research at the molecular level will increase significantly. Already, a comparison between the oat and wheat genomes showing a lower number of genes encoding gluten-like proteins in the oat genome has been published [54]. Understanding oat on a genetic level will help to improve the nutritional quality and agronomic traits of oat.

This study aimed to show the complexity of the metallothionein gene family in oat. We assume that these Cys-rich proteins with antioxidant properties are of key importance during plant growth and development, not only in favorable conditions but also in stress conditions caused by the presence of heavy metals. We identified 21 *MT* genes in the oat genome and analyzed their structure, evolution, and chromosomal localization. Moreover, we investigated the presence of cis-regulatory elements in the promoters of the *AsMT* genes. To verify the potential role of AsMTs in oat development and stress response, we examined the expression of *AsMT* genes during germination and in seedlings grown in the presence of Zn, Cd, and a mixture of Zn and Cd in a hydroponic culture.

## 2. Materials and Methods

### 2.1. Oat Metallothionein Genome-Wide Identification and Analysis of Putative AsMT Proteins

To identify the oat *MT* genes (*AsMT*), the *MT* sequences from *Hordeum vulgare* L., *O. sativa*, and *Z. mays* (downloaded from NCBI’s GeneBank) were used as queries to search against the hexaploid oat genome OT3098 v2 [53] via the Grain Genes database (https://wheat.pw.usda.gov/, accessed on 25 April 2022). Default parameters for BLAST search were used. The putative *AsMT* gene family members were downloaded and verified using the Pfam database (http://pfam.xfam.org/, accessed on 25 April 2022). The theoretical molecular weight (MW) and isoelectric point (pI) of putative AsMTs were calculated using the ProtParam program (http://web.expasy.org/protparam/ accessed on 26 April 2022). Subcellular localizations were predicted using the Plany4t-mPLoc server (http://www.csbio.sjtu.edu.cn/bioinf/plant-multi/#, accessed on 5 May 2022) [55]. The Gene Structure Display Server (GSDS, http://gsds.gao-lab.org/, accessed on 5 May 2022) was used to compare the coding sequences (CDS) with the corresponding genomic DNA (gDNA) sequences of oat AsMTs downloaded from the Grain Genes database [56]. Data regarding the chromosome localization of *AsMT*s were downloaded from the Grain Genes database and analyzed using MG2C (http://mg2c.iask.in/mg2c_v2.1/, accessed on 6 May 2022).

### 2.2. Phylogenetic Analysis and Conserved Motif Analysis

The amino acid sequences of 21 AsMTs and MTs from *A. thaliana* (AtMT), *Z. mays* (ZmMT), and *O. sativa* (OsMT) were aligned, and a phylogenetic tree was constructed using MEGA-11 software (version 11.0.13) with a bootstrap of 1000 replicates [57]. The sequences of MT protein from *A. thaliana*, *O. sativa*, and *Z. mays* were downloaded from the Ensembl database (https://plants.ensembl.org/, accessed on 28 April 2022).

Multiple sequence alignments of type-1–4 AsMTs and MTs from other plants were constructed using MEGA-11 software (version 11.0.13). MT sequences from other plants were obtained from the NCBI database. The online tool MEME (ver. 5.4.1, https://meme-suite.org/meme/tools/meme, accessed on 6 May 2022) was used to find conserved motifs in the 21 AsMT amino acid sequences, with the maximum motif number set to 5.

### 2.3. Prediction of Cis-Responsive Elements in AsMT Promoters

A 1500 bp fragment of the genomic region upstream of the start codon (ATG) was obtained from the Grain Genes database and used to search for the cis-acting regulatory elements (CREs) using the PlantCARE database (http://bioinformatics.psb.ugent.be/webtools/plantcare/html/, accessed on 25 May 2022). The positions of the CREs were marked in the diagram using TBtools (https://bio.tools/tbtools, accessed on 25 May 2022, version 1.123).

### 2.4. Plant Material

Seeds of oat cv. Bingo, purchased from Plant Breeding Strzelce Ltd., PBAI Group, Strzelce, Łódź Voivodeship, Poland, were used for experiments. The seeds were sterilized using a mixture of 96% ethanol and 30% H_2_O_2_ (1:1, *v*/*v*) for 1 min and rinsed five times in sterile distilled water.

For the analysis of *AsMT1-4* expression during germination, sterilized seeds were placed in Petri dishes on filter paper soaked in 3 mL of sterile distilled water. The Petri dishes were incubated in darkness at 23 °C. After 3, 6, 9, 12, 24, and 48 hours, 10 seeds were collected and frozen in liquid nitrogen and kept at −80 °C for further analysis. Dry, non-germinating seeds were used as a reference sample (0 h). The experiment was repeated 3 times.

For the analysis of *AsMT1-4* expression under heavy metal stress, sterilized seeds were germinated for 4 days in Petri dishes lined with filter paper that was moistened with 3 mL of sterile distilled water. Four-day-old seedlings of similar size were placed in 1000 mL plastic vessels containing Hoagland medium [58] and maintained in a hydroponic culture in a growth chamber at 21 ± 2 °C under a light intensity of 100 μmol m^−2^ s^−1^ (16/8 h light/dark). After 3 days of acclimation, stress was induced by changing the medium to Hoagland’s medium amended with 200 µM ZnSO_4_, 100 µM CdSO_4_, or a mixture of 200 µM ZnSO_4_ and 100 µM CdSO_4_. The medium was aerated consistently using an air pump to avoid hypoxia (Hailea ACO-2201, Happet, Poznań, Poland). After 3, 7, and 14 days of treatment, the length of oat shoots and roots and their fresh and dry biomass were measured. Moreover, roots and shoots were washed 3 times in sterile distilled water, frozen in liquid nitrogen, and kept at −80 °C for further analyses.

### 2.5. Total RNA Isolation

Plant tissues were ground in liquid nitrogen using a mortar and pestle. For isolating the RNA from shoots and roots, 0.1 g of tissue was used, and the RNA was isolated using an RNeasy Plant Mini Kit (QIAGEN, Hilden, Germany). For the seeds, 0.2 g of ground tissue was used for RNA isolation according to the protocol described by Wang et al. [59], with some modifications. The changes to the protocol included a larger volume of RNA extraction buffer (600 µL), a larger volume of 20% sodium dodecyl sulfate (SDS, 30 µL), and longer RNA precipitation in ethanol (overnight at −20 °C). The quality and quantity of isolated RNA were checked via agarose gel electrophoresis and spectrophotometric measurement using a NanoDrop^TM^ Lite Spectrophotometer (Thermo Fisher Scientific, Waltham, MA, USA).

### 2.6. Quantitative Real-Time PCR (RT-qPCR) Analysis

To remove any DNA contamination from the RNA samples, 1 μg of total RNA was treated with 1 U of DNase I (Thermo Fisher Scientific, Waltham, MA, USA) and incubated at 37 °C for 30 min. The cDNA was synthesized from 1 μg of total RNA using a mixture of 2.5 μM oligo(dT)_20_ primer and 0.2 μg of random hexamers with an NG dART RT Kit (EURx, Gdańsk, Poland), according to the manufacturer’s protocol. The reaction was performed at 25 °C for 10 min, followed by 50 min at 50 °C. The cDNA was stored at −20 °C. The quality of the cDNA was checked via RT-PCR. The PCR mixture contained 2 μL of 10x Pol Buffer B, 0.2 mM of dNTPs, 0.3 μM of forward and reverse AsACT primers (Table 1), 1.25 U of Opti*Taq* DNA Polymerase (EURx, Gdańsk, Poland), and 1 μl of cDNA for a total volume of 20 μL. The thermal cycling conditions were as follows: 94 °C for 5 min, 30 cycles of 94 °C for 45 s, 55 °C for 45 s, and 72 °C for 40 s, followed by 72 °C for 7 min.

The RT-qPCR reaction mixture included 4 μL of 1/5 (seeds) or 1/30 (shoots and roots) diluted cDNA, 0.5 μM of gene-specific primers (Table 1), and 5 μL of LightCycler 480 SYBR Green I Master (Roche, Penzberg, Germany) for a total volume of 10 μL. *EIF4A* (Eukaryotic Initiation Factor 4A-3) was used as a reference gene [60]. The reactions were performed in three technical replicates in a LightCycler 480 Instrument II (Roche, Penzberg, Germany). The thermal cycling conditions were as follows: 95 °C for 5 min, 95 °C for 10 s, 60 °C for 20 s, 72 °C for 20 s, over 45 cycles. The SYBR Green I fluorescence signal was recorded at the end of the extension step in each cycle. The specificity of the assay was confirmed by the melt curve analysis, i.e., increasing the temperature from 55 to 95 °C at a ramp rate of 0.11 °C/s. The fold change in gene expression was calculated using LightCycler 480 Software, release 1.5.1.62 (Roche, Penzberg, Germany).

### 2.7. Determination of Photosynthetic Pigments

The content of photosynthetic pigments (chlorophyll a and b, and carotenoids) in oat shoots exposed to heavy metal stress was measured via spectrophotometric measurement using the Epoch Take 3 microplate reader (Agilent BioTek, Santa Clara, CA, USA). One hundred milligrams of plant material was ground in liquid nitrogen, and pigments were extracted with 1 mL of 80% ethanol. The samples were shaken for 15 min (180 rpm) at room temperature and then centrifuged (13,000× *g*, 10 min, room temperature). The absorbance of the supernatant was measured at λ = 470 nm, λ = 648 nm, and λ = 664 nm (Epoch Take 3 microplate reader, Agilent BioTek, Santa Clara, CA, USA). Concentrations of chlorophyll a, chlorophyll b, and carotenoids were calculated according to Lichtenthaler and Wellburn [61].

### 2.8. Determination of Total Phenolic Content

The total phenolic content (TPC) was determined spectrophotometrically using the method described by Singleton and Rossi [62]. Phenolic compounds were extracted from 100 mg of plant material that had been ground in liquid nitrogen (shoots and roots separately) in 1 mL of 80% ethanol. The samples were shaken for 15 min (180 rpm) at room temperature and then centrifuged (13,000× *g*, 10 min, room temperature). The reaction mixture consisted of 500 µL of distilled water, 100 µL of ethanolic plant extract, 250 µL of 25% Na_2_CO_3_, and 125 µL of Folin–Ciocalteau reagent (diluted with distilled water 1:1, *v*/*v*, before use). The samples were incubated for 15 min at room temperature and briefly centrifuged, and the absorbance was measured at λ = 760 nm (Epoch Take 3 microplate reader, Agilent BioTek, Santa Clara, CA, USA). TPC was expressed as micrograms of gallic acid (GA) per gram of FW of plant tissue. 

### 2.9. Determination of Antioxidant Capacity

To extract antioxidants from the shoots and roots of heavy-metal-stressed oat plants, 300 mg of ground tissue was mixed with 5 mL of 50% methanol (*v*/*v*). The samples were shaken for 20 min (250 rpm) at room temperature and then centrifuged (15 min, 5000× *g*, 4 °C). The supernatant was collected, and the extraction procedure was repeated twice. The extracts obtained from each extraction step were mixed and subjected to further analysis.

#### 2.9.1. ABTS Assay

The mixed-mode ABTS (2,2′-azino-bis(3-ethylbenzothiazoline-6-sulfonic acid)) method for determining the quantity of hydrophilic and lipophilic antioxidants was performed according to Re et al. [63]. An ABTS radical cation (ABTS^●+^) was produced during the reaction of a 7 mM solution of ABTS with 2.45 mM of potassium persulfate at a ratio of 2:1 (*v*/*v*) overnight in darkness. Before use, the ABTS^●+^ solution was diluted with ethanol to an absorbance of 0.7 (±0.02) at λ = 734 nm. Next, 100 µL of plant methanolic extract was added to 150 µL of ABTS^●+^ solution and the mixture was incubated at 30 °C for 5 min. The absorbance was measured at λ = 734 nm (Epoch Take 3 microplate reader, Agilent BioTek, Santa Clara, CA, USA), and the antioxidant capacity (AC) was expressed as a water-soluble analog of vitamin E Trolox (TE, 6-hydroxy-2,5,7,8-tetramethylchromane-2-carboxylic acid) equivalents (µmol TE per 1 g fresh weight of plant tissue). The calibration curve, %ABTS = (166.03 ± 0.53)c_TE_ + (6.51 ± 0.30), was prepared using working solutions of TE in methanol between 0.01 and 0.15 μmol/mL.

#### 2.9.2. Ferric Reducing Antioxidant Power (FRAP) Assay

To quantify the hydrophilic antioxidants in the oat seedlings, the single electron transfer (SET) method, i.e., a FRAP assay, was performed according to the procedure originally developed by Benzie and Strain [64], with some modifications. The FRAP solution contained 100 mL of 0.1 M acetate buffer (pH 3.6), 10 mL of a 10 mM TPTZ (2,4,6-tris(2-pyridyl)-s-triazine) solution in 40 mM HCl, and 10 mL of 20 mM FeCl_3_. Before usage, it was incubated at 40 °C for 15 min. The reaction mixture contained 50–100 µL of plant methanolic extract, 100 µL of FRAP solution, and distilled water to a final volume of 250 µL. The samples were incubated for 20 min in darkness. The absorbance was measured at λ = 593 nm (Epoch Take 3 microplate reader, Agilent BioTek, Santa Clara, CA, USA), and the AC was expressed as TE equivalents (µmol TE per 1 g FW). Calibration curves were prepared using working solutions of TE in methanol between 1.00 × 10^−3^ and 1.70 × 10^−2^ μmol/mL. The least squares method was applied to calculate the line’s equation: A_593_ = (51.51 ± 0.42)c_TE_ + (0.023 ± 0.004) resulting in R^2^ = 0.9997.

### 2.10. Statistical Analysis

The results are expressed as mean values, and error bars represent the standard deviation (SD). Before the statistical assessment, data normality was tested using the Shapiro–Wilk test. The statistical analysis of the experimental data was performed via one-way analysis of variance (ANOVA) followed by a post-hoc Tukey’s test. Pearson correlations were calculated to demonstrate the relations among the measured traits. The programs Microsoft Excel, Past 4.0 [65], RStudio [66], and Phyton were used for calculations and the preparation of graphs.

## 3. Results

### 3.1. Identification and Chromosome Distribution of A. sativa Metallothionein (AsMT) Genes

**Figure 1:**
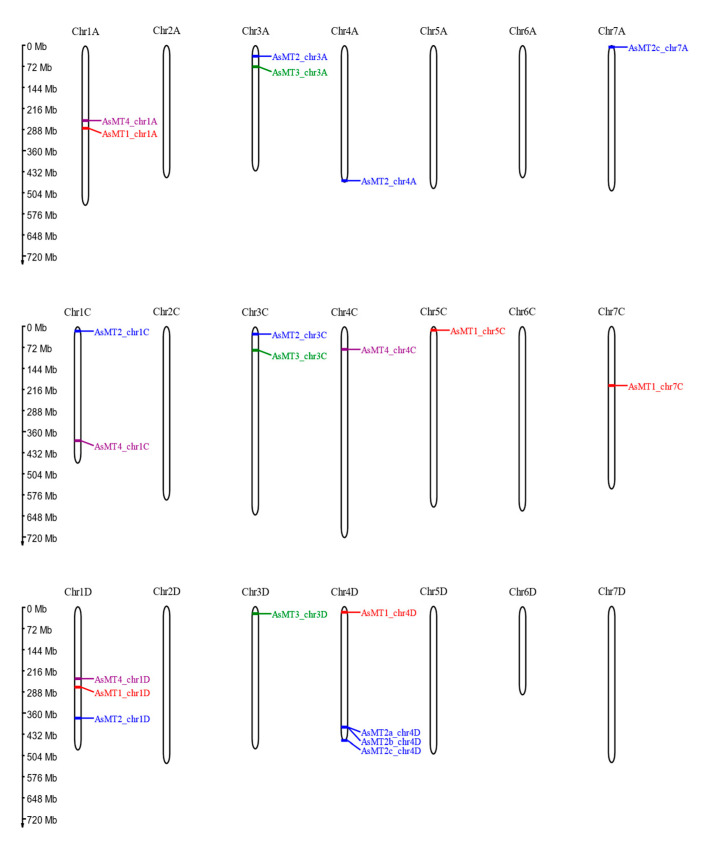
Chromosome localization of oat *MT* genes. Type-1 metallothioneins are labelled in red, type-2 *pMTs* in blue, type-3 *pMTs* in green, and type-4 *pMTs* in purple.

BLAST screening of the oat genome revealed the presence of 21 genes that encode putative oat MTs. *AsMT* genes were located in 12 out of the 21 oat chromosomes (Figure 1). The type of putative protein encoded by the identified genes was at this stage determined by the homology to the MT sequence used as a query in the BLAST search, to be further confirmed by an in silico analysis of putative amino acid sequences and phylogenetic analysis. The identified genes were named according to the encoded MT type (MT1-4) and chromosome localization (chr1-7, A, C, D). The highest number of *MT* genes were located in the subgenome D, which accounted for eight genes, whereas seven genes were present in the subgenome C, and six genes in the subgenome A. With some exceptions, the number and chromosomal localization of *AsMT1-4* differed among groups of chromosomes. On chromosomes 3A and 3C, nearby genes encoding AsMT2 and AsMT3 were present, whereas on chromosome 3D, only the *AsMT3* gene was present. On chromosomes 1A and 1D, the *AsMT1* and *AsMT4* genes were located close to one another. On chromosome 1C, the *AsMT4* gene was present, although not in the same locus (Figure 1). The majority of *AsMTs* were located on the distal parts of chromosomes; only *AsMT1_chr1A*, *AsMT4_chr1A*, *AsMT1_chr7C*, *AsMT1_chr1D*, and *AsMT4_chr1D* were located in the central part of chromosomes. In all three subgenomes, only chromosomes 2 and 6 had no *MT* genes (Figure 1).

**Figure 2:**
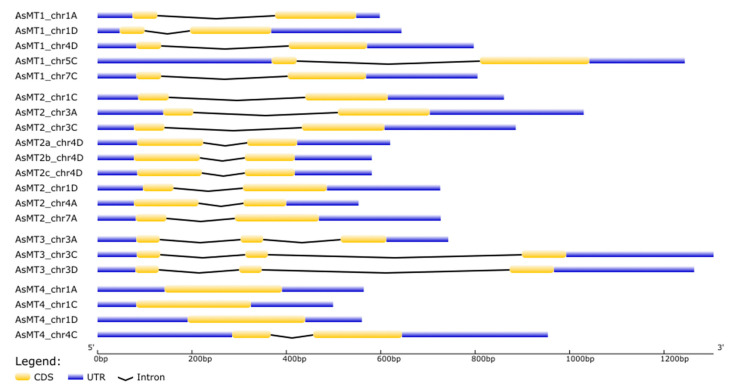
Intron–exon structure of oat *MT* genes. Coding sequences (CDS), untranslated regions (UTR), and introns are displayed as yellow boxes, blue boxes, and black lines, respectively.

The length of the introns and exons of the *AsMT* nucleotide sequences was very variable and ranged from 243 bp (*AsMT4_chr1C*) to 910 bp (*AsMT3_chr3C*) in length (Figure 2, Table 2). Type-1 and -2 *AsMTs* had one intron, type-3 *AsMTs* had two introns, and type-4 *AsMTs* had no introns, except for *AsMT4_chr4C*, which had one intron (Figure 2).

Further in silico analysis of the putative amino acid sequences of AsMT1-4 was performed to confirm the type of pMTs based on the number and distribution of Cys residues (Table 2). It was shown that five AsMTs were type-1 pMTs, nine were type-2 pMTs, three were type-3 pMTs, and four were type-4 pMTs. Atypical numbers and distributions of cysteines were also revealed for some type-1 and -2 AsMTs. AsMT1_chr1A had 11 cysteines, and AsMT1_chr7 had 13 cysteines, instead of the typical 12 cysteines present in type-1 pMTs. AsMT2C_chr4A had 15 cysteines and AsMT2C_chr1D, AsMT2C1_chr4D, AsMT2C2_chr4D, AsMT2C3_chr4D, and AsMT2C_chr7A had 17 cysteines instead of the 14 Cys that is a typical number for type-2 pMTs. For AsMT3 and AsMT4, all analyzed sequences had a typical number of cysteines, i.e., 10 and 17, respectively. The distribution of cysteines was observed in pMTs in other plant species, i.e., two Cys-rich domains for pMT1-3 and three Cys-rich domains for pMT4 (Table 2). Cysteines in MTs are arranged in typical motifs, which were also present in AsMT1–4 (Table 2). The length of the putative amino acid sequences of AsMT ranged from 63 (AsMT3_chr3C and AsMT3_chr3D) to 89 (AsMT4_chr4C), and the molecular weights ranged from 6.66 kDa for AsMT3_chr3C to 8.62 kDa for AsMT4_chr4C. The pI values of putative oat proteins ranged from 4.71 for AsMT2_chr3C to 7.36 for AsMT4_chr1A, AsMT4_chr1C, and AsMT4_chr1D (Table 2). The results of subcellular localization prediction showed that 11 AsMTs had a single subcellular localization in either cytoplasm or nuclei. For other AsMTs, multiple subcellular localizations were predicted, i.e., the cytoplasm, nuclei, cell membrane, and chloroplasts (Table 2). 

### 3.2. Analysis of Conserved Motifs in the Amino Acid Sequences of AsMTs

**Figure 3:**
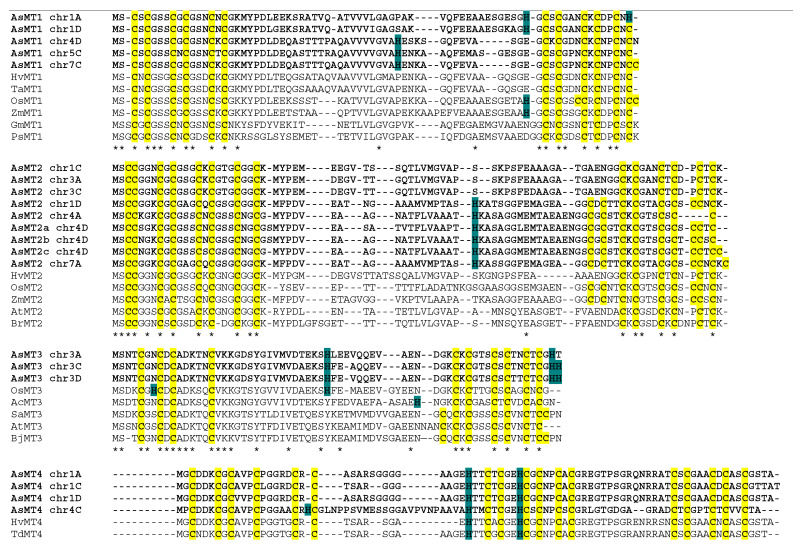

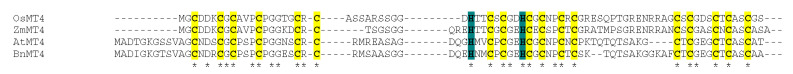
Amino acid alignments of the representative members of each type of pMT. Cysteine residues are highlighted in yellow, histidine residues are highlighted in blue, AsMTs are marked in bold, and asterisks (*) indicate identical amino acids. The GenBank accession numbers are as follows: HvMT1 (*Hordeum vulgare* MT1, XP_044981033.1), TaMT1 (*Triticum aestivum*, NP_001392631.1), OsMT1 (*Oryza sativa*, NP_001391526.1), ZmMT1 (*Zea mays*, PWZ25072.1), GmMT1 (*Glycine max*, NP_001359044.1), PsMT1 (*Pisum sativum*, BAD18382.1), HvMT2 (*H. vulgare*, XP_044974743.1), OsMT2 (*O. sativa*, NP_001384880.1), ZmMT2 (*Z. mays*, ACG26701.1), AtMT2 (*Arabidopsis thaliana*, NP_195858.1), BrMT2 (*Brassica rapa*, XP_009125444.1), OsMT3 (*O. sativa*, A2Y1D7.1), AcMT3 (*Ananas comosus*, OAY84410.1), SaMT3 (*Sinapis alba*, KAF8083573.1), AtMT3 (*A. thaliana*, NP_566509.1), BjMT3 (*Brassica juncea*, KAG2309813.1), HvMT4 (*H. vulgare*, KAI5017801.1), TdMT4 (*Triticum dicoccoides*, XP_037426553.1), OsMT4 (*O. sativa*, AAS78805.1), ZmMT4 (*Z. mays*, NP_001105499.1), AtMT4 (*A. thaliana*, NP_001189730.1), BnMT4 (*B. napus*, CAF1701889.1).

The alignments of the amino acid sequences of the AsMT proteins showed that sequences belonging to the same type of pMT are highly conserved even in evolutionarily distant species (Figure 3). These alignments confirmed that identified AsMT proteins belong to respective types of pMTs. A comparison of AsMTs with MTs from other plant species showed that Cys-rich domains are the most conserved parts of pMT sequences in terms of the number and arrangement of cysteines. Moreover, in AsMT4, there were two highly conserved His residues, which were also present in other representatives of this type of pMTs. Interestingly, some of the AsMT4 proteins exhibited atypical features that were not present in pMTs from other plant species. For example, in AsMT1_chr1A, the last Cys is substituted with His, whereas in AsMT4_chr4C there is another, third His within the first Cys-rich domain. Moreover, in AsMT1_chr4D, AsMT1_chr5C, and AsMT1_chr7C, an additional His located within the Cys-free stretch is present. The additional His within this region is also present in six of the type-2 AsMTs and all of the type-3 AsMTs; however, for type-3 pMTs, this additional His is also present in OsMT3. All AsMT3 proteins contain histidine/s at the C-terminus of the amino acid sequence (Figure 3). 

**Figure 4:**
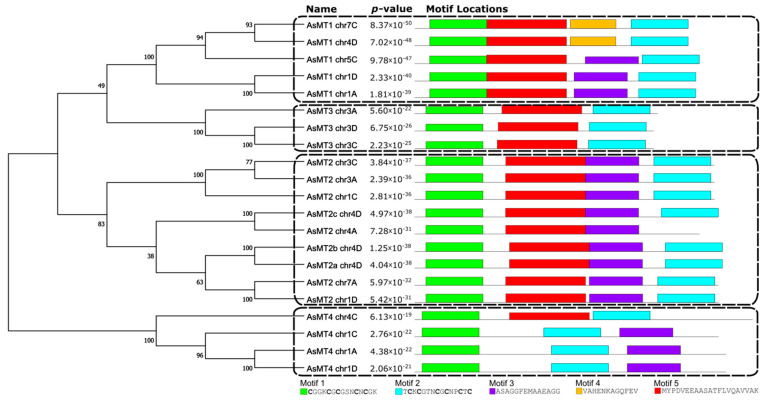
A neighbor-joining cladogram of AsMTs and five identified de novo motifs in oat MTs. Motifs were found using the MEME tool and are marked by different colors. The amino acid sequence of each motif is shown at the bottom of the figure.

The MEME program was used to find in AsMT proteins de novo motifs that could be important for activity or the proper folding of proteins (Figure 4). Five different motifs were identified, and among them, only motifs 1 and 2 (containing Cys residues) were present in all 21 AsMT proteins. Motif 1 is present as the first motif on the N-terminus of the protein, whereas motif 2 is the last one at the C-terminus, except in the case of three type-4 AsMTs, where it was second-to-last. Motif 3 was present in all type-2 AsMTs, in three type-1 AsMTs, and in one type-4 AsMT. Motif 4 was only found in two type-1 MT sequences (AsMT1_chr7C and AsMT1_chr4D). Motif 5 was found in all sequences except for three AsMTs belonging to type-4 (Figure 4). The variation in the occurrence of motifs may be related to the functional divergence of AsMTs.

**Figure 5:**
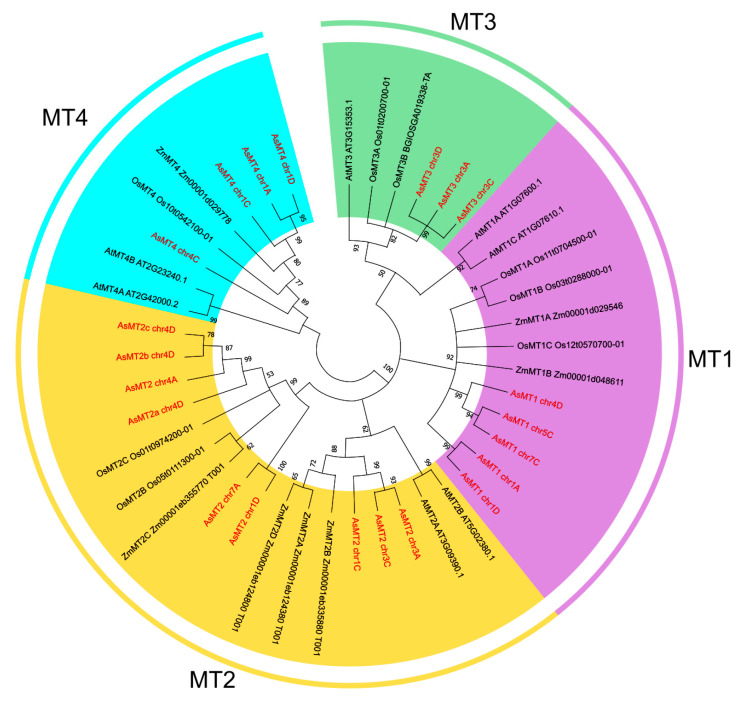
A phylogenetic tree based on the amino acid sequences of *Avena sativa*, *Arabidopsis thaliana, Zea mays*, and *Oryza sativa* MTs. The amino acid sequences were aligned by MEGA11 using the MUSCLE method, and the phylogenetic tree was built using the neighbor-joining method. The four MT types are highlighted in different colors and AsMTs are in red font.

A phylogenetic analysis of MT proteins from selected plant species showed the presence of four separated MT groups and confirmed that identified *AsMT* genes encode MTs belonging to the respective types of pMTs (Figure 5). AsMT2 proteins could be divided into three subgroups showing higher similarity to MT2 from other plant species than to each other. Moreover, one of the type-4 AsMTs is different from the other three AsMT4 proteins (Figure 5). These observations might reflect the polyploidy nature of *A. sativa* genome.

### 3.3. Prediction of Cis-Responsive Elements in AsMT Promoters

**Figure 6:**
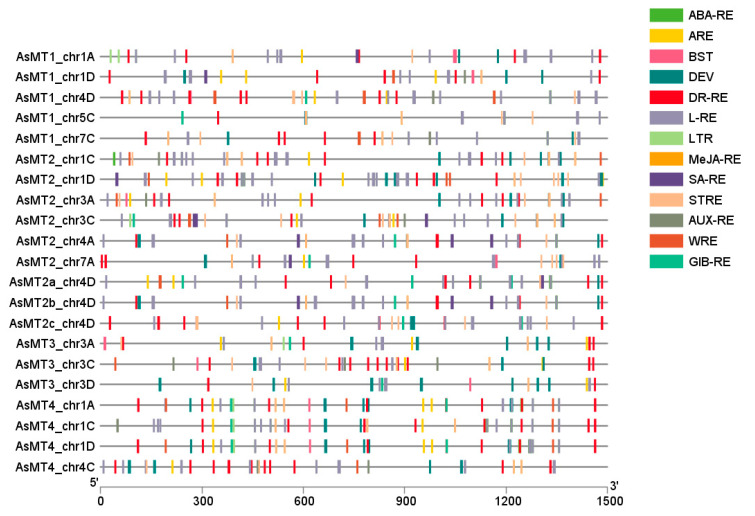
Schematic depiction of predicted cis-acting elements in *AsMT* promoters. The *cis*-acting elements are represented by different colored boxes. The scale at the bottom represents the length of the analyzed sequence. The abbreviations are as follows: ABA-RE—abscisic acid response, ARE —anaerobic induction, BST—biotic stress response, DEV—development-related, DR-RE—drought response, L-RE—light response, LTR—low-temperature response, MeJA-RE—methyl jasmonate response, SA-RE—salicylic acid response, STRE—stress response, AUX-RE—auxin response, WRE—wounding response, GIB-RE—gibberellin response.

A 1500 bp region upstream of the ATG codon for all *AsMT* genes was analyzed using PlantCARE (Figure 6, Tables S1 and S2). Numerous cis-acting elements involved in phytohormone responses, stress reactions, pathogen defense, and development were found. The most common were elements involved in the response to phytohormones and abiotic stress (Table S2). Hormone-responsive elements, predominantly abscisic acid response elements, were found in all *AsMTs*. Regulatory elements related to the response to methyl jasmonate were found in 19 of the 21 analyzed promoters, whereas elements related to the response to gibberellins, salicylic acid, and auxins were less common (Table S1). Among abiotic-stress-responsive elements, the most common were drought response regulatory elements (Table S1). Other common elements were involved in light response and development, whereas the less common elements were those related to the response to biotic stress (Table S2).

### 3.4. AsMT1-4 Expression during Seed Germination

**Figure 7:**
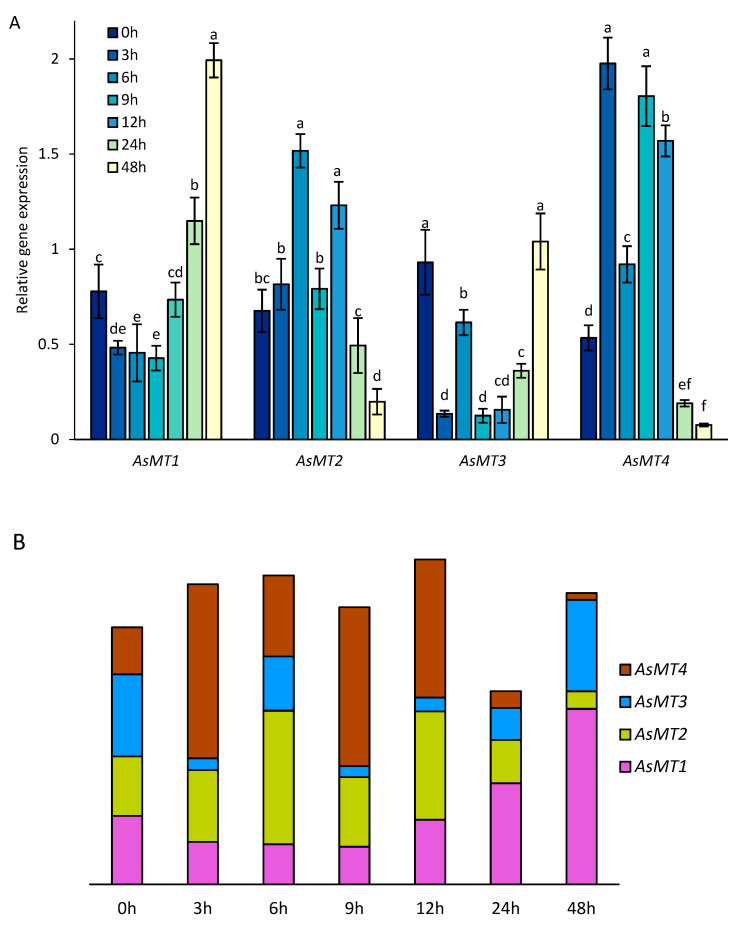
Levels of oat *AsMT1-4* transcripts in germinating seeds. (**A**) Relative expression of *AsMT1-4* in dry seeds (0 h) and during seed germination (3–48 h (hours after the start of germination)). Bars represent the means of three independent experiments ± SD. Values marked with different letters differ significantly (ANOVA, Tukey’s test, *p* < 0.05). (**B**) A schematic representation of the number of *AsMT1-4* transcripts in dry (0 h) and germinating seeds (3–48 h).

Seed germination is the first crucial step in plant growth and is significantly influenced by various environmental factors. Based on the promoter analysis, it might be predicted that AsMTs are involved in various developmental processes including germination. To evaluate the role of MTs during oat germination, the expression of four selected *AsMT* genes representing four types of pMT was determined (Figure 7). The expression of oat *MT* type 1 during the first hours of germination (3–9 h) decreased, but 12 h after the start of germination, *AsMT1* expression increased. Similarly, the expression of *AsMT3* was the highest in the dry seeds 48 h after the start of germination. An inverse trend was observed for *AsMT2*, where the expression peaked after the sixth and twelfth hour of germination, and its level was the lowest at the end of the experiment. The expression of *AsMT4* was the lowest after the forty-eighth hour of germination and the highest after the third and nineth hour (Figure 7A). The total number of *AsMT1-4* transcripts remained relatively constant throughout the analyzed period of germination, except during the 24th hour of germination. The relative amount of each AsMT in the dry and germinating oat seeds was variable throughout the analyzed period (Figure 7B).

### 3.5. Effect of Heavy Metal on Oat Seedling Growth

**Figure 8:**
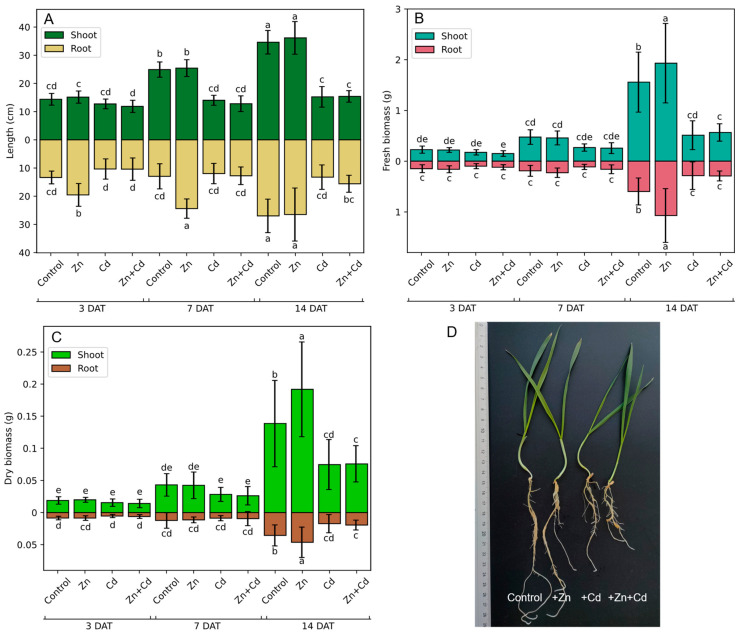
Shoot and root (**A**) length and the (**B**) fresh, and (**C**) dry biomass of oat seedlings subjected to heavy metal stress induced via the application of 200 µM ZnSO_4_, 100 µM CdSO_4_, and 200 µM ZnSO_4_ + 100 µM CdSO_4_. The control comprised non-stressed plants. Bars represent the mean values of measurements of 30 seedlings ± SD (*n* = 3). Values marked by different letters differ significantly (ANOVA, Tukey’s test, *p* < 0.05). (**D**) Photo of oat seedlings after 14 days of stress. DAT—days after treatment.

To assess the effect of heavy metals on oat seedling growth and development, the seedlings were treated with metal ions in hydroponic culture for 14 days (Figure 8). After 3 days of stress treatment, the length (Figure 8A) and biomass (Figure 8B,C) of oat shoots and roots were the same as for seedlings grown in the control conditions, with the only exception being the longer roots of Zn-treated plants; however, there were no differences in the root biomass. After 7 days of stress, there were no significant changes in either the fresh (Figure 8B) or dry (Figure 8C) biomass of the shoots and roots. However, the roots but not the shoots of Zn-treated plants were significantly longer than the roots of control seedlings, whereas the shoots but not the roots of Cd- and Zn + Cd-treated plants were shorter than the control (Figure 8A). After 14 days of heavy metal treatment, there were no differences between the root and shoot lengths of control and Zn-treated plants, but the fresh and dry biomass of the Zn-treated seedlings was higher. Treating plants with Cd and Zn + Cd for 14 days significantly shortened shoots and roots (Figure 8A,D) and reduced the biomass (Figure 8B,C).

### 3.6. Chlorophyll a and b and Carotenoid Content in Response to Heavy Metal

**Figure 9:**
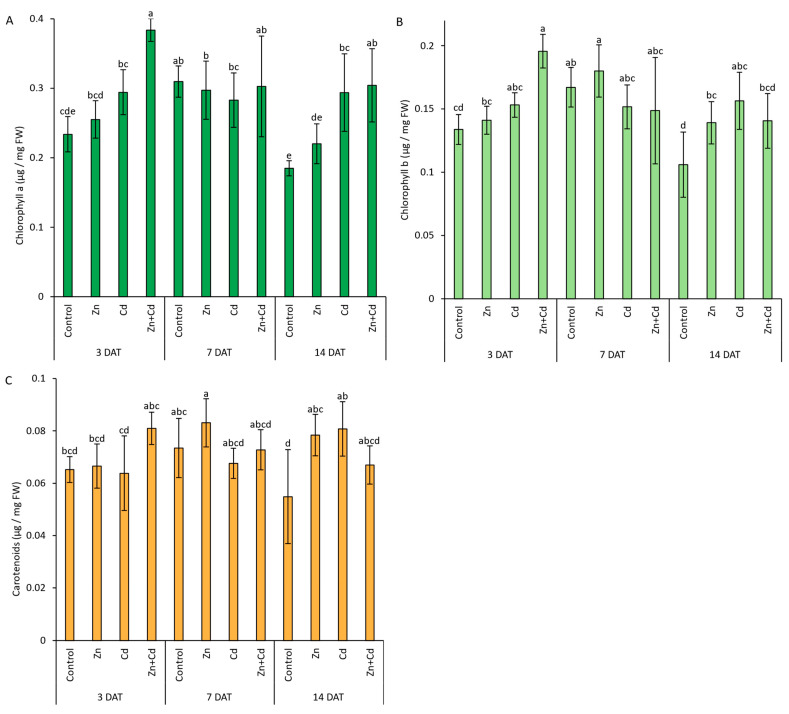
The content of photosynthetic pigments (**A**) chlorophyll a, (**B**) chlorophyll b, and (**C**) carotenoids in oat seedlings subjected to heavy metal stress induced via the application of 200 µM ZnSO_4_, 100 µM CdSO_4_, and 200 µM ZnSO_4_ + 100 µM CdSO_4_. The control comprised non-stressed plants. Bars represent the mean values of three independent experiments ± SD (*n* = 3). Values marked by different letters differ significantly (ANOVA, Tukey’s test, *p* < 0.05). DAT—days after treatment.

The level of chlorophyll a increased in response to Cd and Zn + Cd treatment after 3 and 14 days in comparison to control plants. The highest level of chlorophyll a was observed in Zn + Cd-treated plants after 3 days and was 1.6 times higher than in control plants. Interestingly, after 7 days of treatment, the content was similar across all experimental variants (Figure 9A). The highest level of chlorophyll b was detected in Zn + Cd-treated plants after 3 days of treatment and was around 1.5 times higher than in control plants. After 7 days of treatment, the content of chlorophyll b was similar across all experimental variants, whereas after 14 days of treatment, it increased in Zn- and Cd-treated plants in comparison to the control (Figure 9B). The level of carotenoids remained relatively consistent during the experiment, except that after 14 days of treatment the carotenoid level in the control was lower than that in Zn- and Cd-treated plants (Figure 9C). In the control and Zn-treated plants, the levels of chlorophyll a and b and carotenoids peaked on day 7. In Cd-treated plants, the levels of chlorophyll a and b remained the same throughout the experiment period, but the levels of carotenoids were slightly higher after 14 days of treatment. A similar situation was observed for Zn + Cd-treated plants, where neither the chlorophyll a nor the carotenoid content differed throughout the experiment, but the level of chlorophyll b increased over the treatment period, reaching its maximum after 14 days of treatment (Figure 9).

### 3.7. Antioxidant Capacity of Oat Seedlings in Response to Heavy Metal

**Figure 10:**
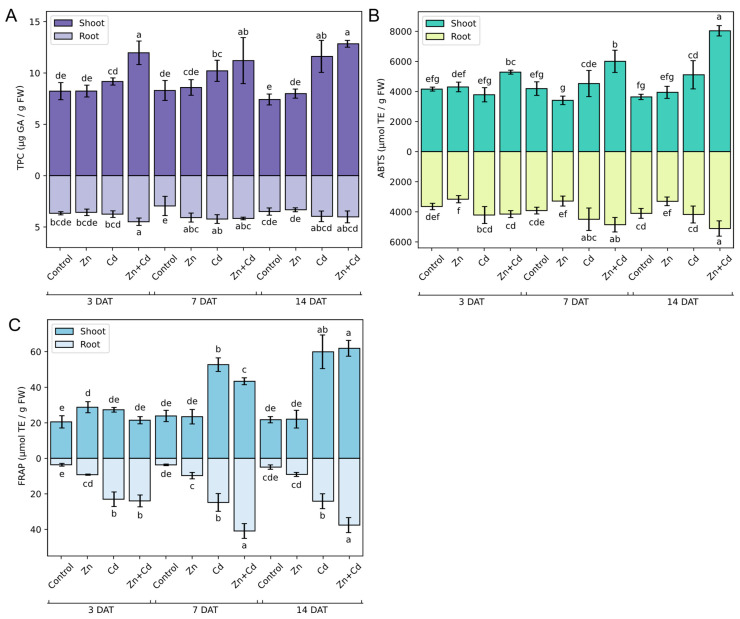
Effect of heavy metal stress induced via the application of 200 µM ZnSO_4_, 100 µM CdSO_4_, and 200 µM ZnSO_4_ + 100 µM CdSO_4_ on the (**A**) total phenolic content (TPC) and antioxidant capacity (**B**,**C**) of oat seedlings shoots and roots, evaluated using (**B**) ABTS and (**C**) FRAP assays. Bars represent the means of three independent experiments ± SD. The control comprised non-stressed plants. Values marked by different letters differ significantly (ANOVA, Tukey’s test, *p* < 0.05). DAT—days after treatment.

To assess the effect of heavy metal stress on the antioxidant properties of oat seedlings, the TPC (Figure 10A) and AC (Figure 10B,C) were determined using the Folin–Ciocalteau, ABTS, and FRAP methods, respectively. The treatment of plants with Zn did not cause a significant difference in TPC in roots and shoots when compared to the control plants. However, on the seventh day of treatment, TPC was around 1.3 times higher in the roots of Zn-treated plants than in control plants (Figure 10A). Treating plants with Cd and Zn + Cd caused an increase in TPC in the shoots of oat seedlings that exceeded that of Cd-treated plants after 3 days of treatment. In the roots of the same plants, TPC increased in plants exposed to Zn + Cd after 3 days of treatment and to Zn and Zn + Cd after 7 days of treatment (Figure 10A).

The antioxidant potential of oat seedlings was measured using the ABTS (Figure 10B) and FRAP (Figure 10C) methods. In general, the AC was not affected by Zn treatment. In the shoots of Zn-treated seedlings after 3 days of treatment, an approximately 1.5 times higher FRAP value than in control seedlings was observed. In the roots of Zn-treated seedlings after 3 and 7 days of treatment, four times higher levels of total hydrophilic antioxidants analyzed by FRAP assay (Figure 10C) were detected compared to the control sample. However, the increase in AC in response to zinc was not observed when using ABTS assay. In fact, in the roots of Zn-treated seedlings, after 14 days of treatment, the ABTS result was lower than for control seedlings (Figure 10B). Treating plants with Cd caused an increase in total antioxidant levels in shoots and roots, but in roots, the high AC was observed on the seventh day after treatment, whereas in shoots, this was observed on the fourteenth day after treatment (Figure 10B). More significant differences between Cd-treated and control seedlings were detected via the FRAP method; i.e., in roots, the AC was higher throughout the experimental period and after 14 days of treatment was 4.9 times higher than in the roots of control seedlings. The FRAP values for shoots of Cd-treated seedlings were higher after 7 and 14 days of treatment, and after 14 days of treatment, the FRAP value was 2.7 times higher than for control seedlings (Figure 10C). Treating plants with the mixture of Zn and Cd had the biggest effect on the AC of oat seedlings. With time, the differences between the Zn + Cd-treated plants and the control plants increased, and on the 14th day, the ABTS values of the Zn + Cd-treated plants were 2.2 and 1.2 times higher for shoots and roots, respectively (Figure 10B). Similarly, the roots of seedlings subjected to Zn and Cd treatment after 3, 7, and 14 days of treatment showed higher FRAP results, and on the 14th day of treatment, the FRAP result was 7.6 times higher than for the control sample. The highest FRAP result was observed for shoots after 14 days of treatment with Zn and Cd, which was 2.8 times higher than in control seedlings (Figure 10C).

### 3.8. AsMT1-4 Expression during Heavy Metal Stress

**Figure 11:**
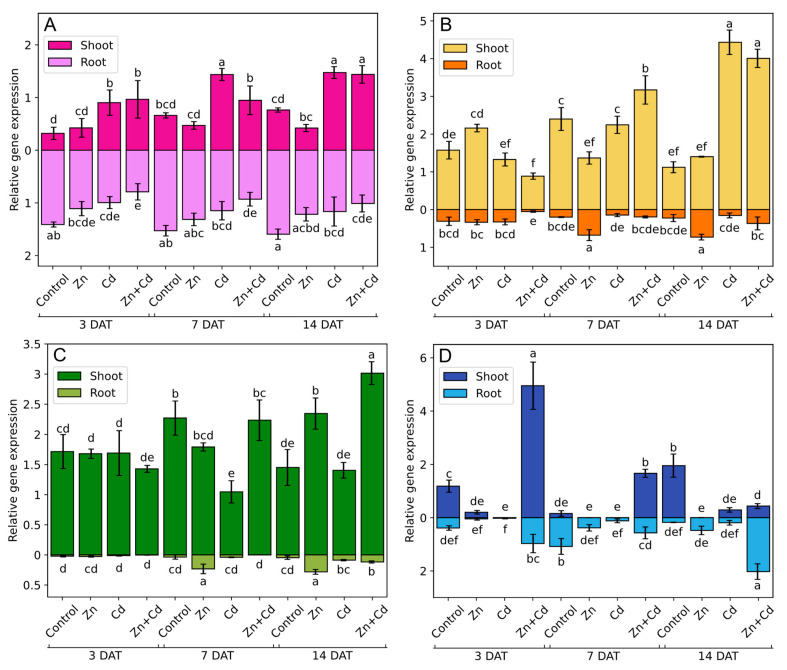
Relative gene expression of (**A**) *AsMT1*, (**B**) *AsMT2*, (**C**) *AsMT3*, and (**D**) *AsMT4* in the shoots and roots of oat seedlings subjected to 14 days of heavy metal stress induced via the application of 200 µM ZnSO_4_, 100 µM CdSO_4_, and 200 µM ZnSO_4_ + 100 µM CdSO_4_. Bars represent the means of three independent experiments ± SD. The control comprised non-stressed plants. Values marked by different letters differ significantly (ANOVA, Tukey’s test, *p* < 0.05). DAT—days after treatment.

To examine the potential role of oat MTs in the response to heavy metals, changes in *MTs* expression were determined after 3, 7, and 14 days of treatment (Figure 11). The expression of *AsMT1* in the roots of Cd- and Zn + Cd-stressed plants was lower than in control conditions; however, in shoots, *AsMT1* expression was generally higher. Zinc treatment did not affect the expression of *AsMT1* (Figure 11A). The expression of *AsMT2* in the shoots of Zn-stressed plants was lower than in the control plants after 7 days of treatment, but after 14 days of treatment, it was on the same level as the expression in the control. However, in roots after 7 days of Zn treatment, the expression of *AsMT2* increased over to three times more than that of the control, and the high expression level lasted until the 14th day of treatment. After 3 days of treatment, the expression of *AsMT2* was the lowest in both the shoots and roots of Zn + Cd-treated plants, but in the following days of treatment, its expression increased in oat shoots, reaching a transcript level over three times higher than in the control on the 14th day of stress (Figure 11B). The expression of *AsMT3* remained unchanged after 3 days of stress induction both in shoots and roots. The first differences in *AsMT3* expression between the control and the heavy-metal-treated plants were detected on the seventh day, where a twofold decrease in expression was observed in the shoots of Cd-treated plants. Moreover, an over sixfold increase in *AsMT3* expression in the roots of Zn-treated plants was observed on the seventh day after treatment, and on the last day of treatment, this high *AsMT3* expression in the roots of Zn-treated plants was accompanied by an increase in expression in shoots. On the 14th day of stress, *AsMT3* expression in the shoots and roots of Zn + Cd stressed plants was 2.0 and 2.5 times higher when compared to control plants (Figure 11C). Treating plants with Zn and Cd lowered *AsMT4* expression when compared to the control, and the transcript level of *AsMT4* in Zn- and Cd-treated plants remained at the same level over the course of treatment. Treating plants with a mixture of Zn + Cd caused a fourfold increase in *AsMT4* expression in shoots and an increase of 2.5 times in roots after just 3 days of stress. Over the following days of stress, the expression level in shoots decreased, and on the 14th day, it became 4.5 times lower than the control plants. However, in the roots of the same plants, i.e., the Zn + Cd-treated plants, *AsMT4* expression increased and was 11 times higher when compared to the control (Figure 11D).

### 3.9. Correlations among AsMT1-4 Gene Expression, the Content of Photosynthetic Pigments, and Antioxidant Content

**Figure 12:**
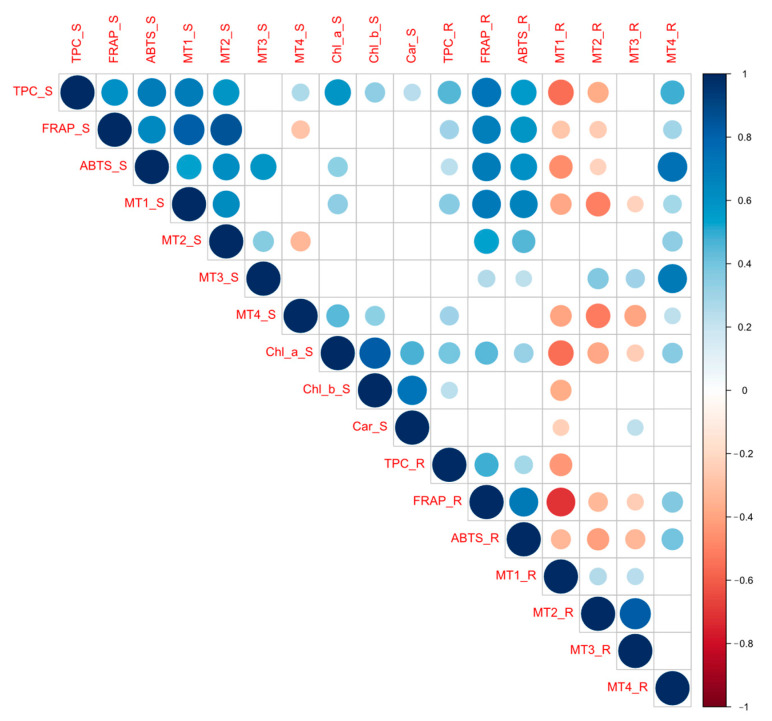
Pearson correlation between *AsMT* gene expression (MT1, MT2, MT3, and MT4), antioxidant capacity (measured using ABTS and FRAP methods), total phenolic content (TPC), and the levels of photosynthetic pigments (chlorophyll a-Chl_a, chlorophyll b-Chl_b, and carotenoids-Car) in shoots (S) and roots (R) of oat seedlings. Only significant correlations are demonstrated (*p* < 0.05).

Pearson correlation analysis (Figure 12) showed high positive correlations between the expression of *AsMT1* and *AsMT2* and TPC and AC (detected using FRAP and ABTS methods) in shoots. The expression of *AsMT3* in shoots had a significant positive correlation with ABTS results. In contrast, negative correlations were noted between the *AsMT1*, *AsMT2*, and *AsMT3* expression and TPC and AC determined via FRAP and ABTS in roots. The expression of *AsMT4* in shoots showed a low but significant positive correlation with TPC, whereas a negative correlation was observed with AC measured via FRAP. In roots, the expression of *AsMT4* correlated positively with the ABTS and FRAP values. In both shoots and roots, TPC and AC correlated positively with each other, and a positive correlation among those parameters was also observed between shoots and roots. The content of photosynthetic pigments correlated positively with TPC and ABTS values. Interestingly, the expression of *AsMT4* in both shoots and roots correlated positively with chlorophyll a and b but not with carotenoids. In contrast, the expression of *AsMT1*, *AsMT2*, and *AsMT3* in roots correlated negatively with photosynthetic pigments. In general, the expression of *AsMT1* and *AsMT2* in roots correlated negatively with parameters measured in shoots (i.e., TPC, AC, Chl a, and Chl b). Positive correlations were also observed between TPC and chlorophyll a, chlorophyll b, and carotenoids. Interestingly, a positive correlation was found between the levels of chlorophyll a and AC measured via ABTS in both shoots and roots. In shoots and roots, the expression of *AsMT2* correlated positively with the expression of *AsMT1* and *AsMT3*, whereas a negative correlation between the expression of *AsMT2* and *AsMT4* in roots was observed. The expression of *AsMT1* and *AsMT4* in shoots correlated negatively with the expression of *AsMT1*, *AsMT2*, and *AsMT3* in roots (Figure 12).

## 4. Discussion

Oat has a complex evolutionary history, which reflects the high number of *MT* genes, of which there are 21. In comparison, *A. thaliana* has a 135 Mb genome and seven *MT* genes [38], *O. sativa* has a 420 Mb genome and eleven *MT* genes [40], and *Z. mays* has a genome size of ~2500 Mb and nine *MT* genes [41]. Previous studies have shown that the number of *MT* genes does not correlate with genome size but with plant ploidy [41]. In the allotetraploid plant *B. napus*, 16 *MT* genes have been identified, and in *Brassica juncea*, 12 *MT* genes have been found. In comparison, in diploid *Brassica rapa*, *Brassica oleracea*, and *Brassica nigra*, eight, nine, and seven *MT* genes have been identified, respectively [42]. The number of introns found in *AsMT* genes is typical for *pMT* genes (data from NCBI’s GenBank) and is rather low since the average number of introns per gene, based on the analysis of monocot rice and dicot *A. thaliana* genomes, is four [67]. In general, in plants, genes that have a less compact structure (i.e., more and longer introns) are expressed at higher levels than those that are more compact [68]. It would be interesting to verify whether the number and length of introns have an impact on the level of expression of oat *MT* genes.

The putative AsMT proteins encoded by the identified genes are very similar to pMTs from other plant species, as confirmed by the amino acid sequence alignments and phylogenetic analysis. However, some AsMTs contain additional His residues that might function in metal binding [14,69]. In general, among pMTs found in angiosperm species, some sequences are characterized by unusual topologies of cysteines and histidines. It is possible that at least some of these non-canonical pMTs are pseudogenes. In MT4B from soybean (*Glycine max* (L.) Merr.), second His is substituted by tyrosine. For this particular protein, it has been shown that the lack of metal-binding His residue results in a lower number of zinc ions (5 Zn^2+^ ions vs. 6 Zn^2+^ ions) that can be bound by this protein [70,71]. The motif-based sequence analysis tool (MEME) results showed that two Cys-rich motifs are conserved in each AsMT [1,72,73]. Moreover, we also identified some other motifs in AsMTs located outside of Cys-rich domains. Similar observations have been made for MTs from *Nicotiana tabacum* L. [13] and *B. napus* [42]. The role of the stretch/linker between Cys-rich domains is not well understood. It has been hypothesized that the linker either allows the protein to fold properly, and metal ions are bound in separate clusters, meaning each Cys-rich domain binds metal ions independently from the other Cys-rich domain, or it allows for the formation of a single metal-binding cluster [12]. Recent experiments on *Cicer arietinum* L. MT2 showed that the linker does not play an important role in protein folding [74]. The presence of conserved motifs outside of Cys-rich domains suggests that the linker region has some physiological functions; however, this needs to be further evaluated. Although MTs are typically viewed as cytosolic proteins, in silico prediction showed that AsMTs could also be localized in the nucleus. The subcellular localizations were shown experimentally for pMTs from different plant species, e.g., rice MT1e in the nucleus [75], *Ziziphus jujuba* Mill. MT1 in the cytosol and the nucleus [76], and type-2 MT from *B. napus* in cytosol when heterologously expressed in yeast cells [77]. Interestingly, proteins smaller than 10 kDa could be transported into the nucleus by passive diffusion, and this kind of transport to the nucleus has been observed for MTII in animals [78]. Moreover, in silico analysis has shown that AsMTs could be localized in the chloroplast and the cell membrane. The membrane localization has not yet been observed for any MTs, but for animal MT, the localization in the intermembrane space of mitochondria has been shown [79]. Therefore, it is possible that in plants, MTs also function in the nucleus and chloroplasts.

The accurate prediction of cis-regulatory elements in promoters remains a challenge for bioinformatics and computational biology; however, this analysis provides valuable insight into the probable functions of proteins encoded by analyzed genes [80]. Based on this analysis, it is highly plausible to hypothesize that oat MTs are involved in stress adaptation and growth and development. As shown previously, the *MT* promoters of other plants, including *Z. mays, O. sativa*, and *A. thaliana*, also have a large number of diversified regulatory elements [2,40,41]. Unfortunately, experimental studies confirming the functionality of in-silico-predicted regulatory elements in *pMTs* are rather scarce. It has been shown that the type-1 rice MT promoter can be induced by wounding, Cu, and PEG treatment [81]. In another study, promoter analysis of type-2 *O. sativa* MT in a transgenic *A. thaliana* plant showed that the promoter activity was affected by phytohormones, PEG, cold, heat, H_2_O_2_, and metals. Although different deletion mutants of the full promoters were generated in this study, their activity under various stresses was not evaluated [82]. A more detailed analysis of heavy-metal-responsive elements has been provided for type-1 MT from rice showing which regions of the promoter are responsible for metal-inducible expression [83]. Most studies focus on the expression of *MTs* under specific stresses, which is only indirect proof of the promoter functionality [13,84,85,86,87].

ROS play dual roles in each living organism; i.e., on the one hand they serve as signaling molecules, and on the other they are toxic and might lead to oxidative stress [88]. Every stress ultimately leads to an increase in the number of ROS in the cells [89]. The expression of *pMTs* is upregulated by a myriad of stress stimuli, and one possible explanation is that pMTs are general stress proteins participating in plant adaptation to a variety of environmental stimuli via ROS scavenging [90]. The reaction of the metaled form of MT with ROS leads to the release of metal ions, which, depending on the type of metal, might have a positive/negative impact on the cell. Moreover, -SH groups of cysteines could be further oxidized, which will lead to the formation of disulfide bridges (the oxidized form of MT). To bind the metal-ion disulfide bridges in MTs need to be reduced [72] by enzymes such as protein-disulfide isomerase (PDI; EC 5.3.4.1) [91]. Seed dormancy and germination are complex processes controlled by ROS [92]. Some studies have demonstrated a clear link between ROS, germination, and pMTs. In magnetoprimed tomato seeds, the level of H_2_O_2_ increased significantly, and the expression of type-1 and type-4 *MTs* increased by around 15 times [93]. *A. thaliana* seeds overexpressing *Nelumbo nucifera* Gaernt. *MT2a* and *MT3* were more resistant to accelerated aging (caused by high-temperature treatment). Although the number of ROS was not determined in those seeds, it was shown that the SOD level was significantly downregulated by accelerated aging and was higher in transgenic seeds than in wild-type seeds, though only after accelerated aging treatment [36]. In our study, we observed that although the expression of oat *MT* genes changed at every investigated stage of germination, the total number of *AsMT* transcripts remained stable throughout the analyzed period. It is plausible to hypothesize that pMTs serve as ROS regulators during germination; however, the lack of detailed studies on pMTs and germination does not allow us to provide a comprehensive picture of the function/s of pMTs in this process.

Zinc is essential for all living organisms; it is a micronutrient involved in almost every conceivable metabolic process [94], whereas cadmium is highly toxic and does not play any physiological roles [95]. Although completely different in terms of function, these two metal ions share similar physiochemical properties, and therefore, cadmium can be uptaken from soil and transported within the plant via zinc transport proteins. The toxicity of cadmium is at least in part due to its interference with zinc homeostasis [96]. An excess of zinc is toxic to plants; however, plants significantly differ in their level of zinc sensitivity. The threshold for zinc toxicity depends on the plant species, the time of treatment, and the composition of the medium. We observed that zinc promoted the growth of oat seedlings, and a similar effect was observed for various plant species [97]. As expected, cadmium significantly reduced the growth of oat seedlings, as has been demonstrated in various plant species. In contrast to our study, one study showed that the application of a mixture of zinc and cadmium did not negatively affect the growth of tomato, whereas the same concentration of cadmium significantly decreased the growth of plants, possibly due to the limiting amount of cadmium that can enter plant roots when both metals are present in the medium [98]. In wheat, the foliar application of zinc also alleviated the negative effects of cadmium on plant growth and yield [99], which is possibly caused by the inability of cadmium to replace zinc in Zn-binding proteins when the number of zinc ions is high. This positive effect of zinc on Cd-treated plants is highly dependent on zinc concentration [100]; therefore, it could be concluded that in our study the concentration of zinc was too high to mitigate the negative effect of cadmium. In various plant species, a decrease in chlorophyll and carotenoid content in Cd-treated plants was observed, e.g., in tomato (*Lycopersicum esculentum* Mill.) [101], pea (*Pisum sativum* L.) [102], and *Salvia sclarea* L. plants [103]. The effect of zinc on the content of photosynthetic pigments is dose-dependent; i.e., a low level of zinc increased the content of pigments, whereas a high level of zinc led to a decrease in pigment content [104,105]. However, here, we observed that the levels of photosynthetic pigments were unaffected by zinc, whereas cadmium elevated the level of pigments, especially chlorophyll a. It might be hypothesized that the zinc concentration was too high to induce the biosynthesis of photosynthetic pigments and too low to induce pigment degradation. The observed induction of pigment biosynthesis by cadmium could be supported by studies on the age-dependent response to Cd stress. For instance, in maize, the increase in chlorophyll a + b content in response to Cd was observed in young leaf segments but not in mature and old ones [106]. Moreover, most plants that are not hyperaccumulators sequester toxic metal ions into root vacuoles and do not translocate them into shoots [107]. Therefore, we hypothesized that in the oat tested in this study, cadmium ions were retained in the root. The observed increase in photosynthetic pigment content is a consequence of signal transduction about stress stimuli rather than the presence of Cd ions in the shoot.

The ABTS assay is suitable for the analysis of both hydrophilic and lipophilic antioxidants. It is a mixed-mode test based on single electron transfer (SET), hydrogen atom transfer (HAT), and proton-coupled electron transfer (PCET) mechanisms [108]. FRAP assay is a SET-based method and allows for the quantification of most hydrophilic antioxidants with a redox potential not lower than that of the redox pair Fe^3+^/Fe^2+^ [109]. The different mechanisms of the used methods and varied affinities toward hydrophobic and hydrophilic antioxidants account for why the AC values measured by the ABTS and FRAP methods differ by two orders of magnitude. Moreover, the content of hydrophilic phenolics as well-known antioxidants [110] was determined. A significant positive correlation among the values obtained by these analyses was observed in this study and has been shown previously [111]. Plant extracts with high values obtained by ABTS assay probably contain more primary antioxidants, i.e., hydrogen or electron donors, whereas those with higher FRAP values might contribute to the higher content of secondary antioxidants (i.e., antioxidants that act indirectly by oxygen scavenging and the chelation of transition metal ions) [109]. Metallothioneins could act as primary antioxidants, because the direct reaction of MT with ROS has been shown [27]. On the other hand, MTs bind to transition metal ions including copper, thus limiting the Fenton and Haber Weiss reactions [112].

The regulation of *MT1-3* expression by heavy metal ions has been demonstrated for a wide range of plant species and different metals [38,85,86,113], although not for oat MTs. The comprehensive analysis of the expression of the *MT* genes representing each type of pMT in one plant species is rather rare in the literature. For example, Ahn et al. [91] showed the variable expression of *Brassica rapa MT1-3* in response to metals; however, in this study, analysis was performed on the whole seedlings. Similarly, in *A. thaliana* seedlings, different levels of regulation of the expression of *MT1-3* by copper was observed [38]. A more detailed analysis of the expression of *MT* genes in response to arsen was performed for *B. napus* [42] and *Z. mays MTs* in response to Cu, Cd, and Pb [41]. Similarly to our results, this research highlights the potential distinct role of each type of pMT depending on the metal ions, plant tissue, and stage of plant development. The expression of type-4 pMTs is restricted to developing and mature seeds and declines rapidly after the start of germination [7,37,41,114]. It has however been shown that in the resurrection plant, *Xerophyta humilis MT4* is upregulated during dehydration and downregulated during rehydration [115], indicating that the role of type-4 pMTs is not limited to seeds. Most studies analyzing the metal-responsiveness of *pMTs* are restricted to vegetative tissue, and thus, knowledge about the expression of *pMT4* in response to various metals is limited. We have previously shown that the expression of *B. napus MT4* is induced by Zn and more significantly by Cd but not by Cu in germinating seeds [69]. The expression of *B. napus MT4* was also regulated by arsen in 7-day-old seedlings [42]. In *A. thaliana* siliques, the expression of *MT4* genes is induced by Cd but not by Cu, Fe, Zn, or Hg [114]. Interestingly, here, we showed that the highest increase in the expression of *AsMT4* was observed when seedlings were treated with a mixture of Zn and Cd, whereas treatment with zinc or cadmium separately did not change or even downregulate *AsMT4* expression. This phenomenon could be explained by the possible role of type-4 pMTs as specificity filters; i.e., due to the presence of His residues, pMT4 can discriminate between essential zinc and toxic cadmium [14,69].

## 5. Conclusions

The widely known hypothesis that there is no single unifying function for all types of pMTs and that each type of pMT might play a different role is supported by our comprehensive in silico and wet-lab analysis of the whole family of oat MTs. The expression of *AsMT1* in shoots was induced by Cd and Zn + Cd but not by Zn, which suggests that AsMT1 plays a role in cadmium detoxification. In roots, the expression of *AsMT2* and *AsMT3* is upregulated by Zn but not by Cd and Zn + Cd, which might implicate the role of these oat MTs in zinc homeostasis in roots. The opposite trend, especially 14 days after treatment, was observed for *AsMT2* expression in shoots, which implies that AsMT2 is responsible for Cd binding in roots. The expression of *AsMT3* in shoots 14 days after treatment was induced by Zn and Zn + Cd but not by Cd, which indicates the role of AsMT3 in zinc homeostasis in shoots, but at later developmental stages. For AsMT4, we propose the role of a zinc specificity filter. Moreover, based on the Pearson correlation analysis, we propose that AsMT1 and AsMT2 play a role in antioxidative response in shoots but not in roots, whereas AsMT4 plays this role in roots but not in shoots. AsMT3 is probably not involved in defense against ROS in oat seedlings, at least when the oxidative stress is induced by heavy metals.

## Figures and Tables

**Table 1:** Sequences of the primers used in this study.

Primer Name	Sequence 5′→ 3′	Product Size [bp]	Target	Reference
AsMT1_qPCR_f AsMT1_qPCR_r	CAAACTGCAAGTGCGGGAAG TTGTTCTCATGAGCCACGCC	103	AsMT1 chr7C	[25]
AsMT2_qPCR_f AsMT2_qPCR_r	CTGCGGAGGGTGCAAGATG AACGATGGCTTGGAAGAGGG	96	AsMT2 chr1C	
AsMT3_qPCR_f AsMT3_qPCR_r	TCCACCATGTCGAACACCTG TGGCTCTTCTCGGTGTCAAC	107	AsMT3 chr3A	
AsMT4_qPCR_f	CACGTGCGGAGAGCACTG	121	AsMT4 chr1D	This study
AsMT4_qPCR_r	ACAGGAGGCGCAGTCACAG			
EIF4A_f EIF4A_r	TCTCGCAGGATACGGATGTCG TCCATCGCATTGGTCGCTCT	88	Eukaryotic Initiation Factor 4A-3	[60]
AsACT_f AsACT_r	CTTCCCCAGTATCGTCGGAC AGGGCAATGTAGGACAGCTT	573	Actin (MH260250.1)	This study

**Table 2:** Characterization of identified *AsMT* genes and putative AsMT proteins. The genes (*AsMT1*_chr7C, *AsMT2*_chr1C, *AsMT3*_chr3A, *AsMT4*_ch1D) that are marked in bold were further analyzed for their expression level (vide infra).

AsMT Type	Gene Name	Gene ID/Position	Strand	gDNA Size (bp)	Protein Length (aa)	MW (kDa)	pI	Cys Number	Cys Sequence Pattern	Length of Spacer between Cys Domains	Predicated Subcellular Localization
MT1	*AsMT1*_chr1A	AVESA.00001b.r3.1Ag0000996	−	474	74	7.45	5.11	11 (6 + 5)	CxC	43	Cyto/Nucl
	*AsMT1*_chr1D	AVESA.00001b.r3.1Dg0000988	+	321	74	7.32	6.50	12 (6 + 6)		43	Cyto/Nucl
	*AsMT1*_chr4D	AVESA.00001b.r3.4Dg0000092	−	489	72	7.26	5.05	12 (6 + 6)		41	Cell mem/Cyto/Nucl
	*AsMT1*_chr5C	AVESA.00001b.r3.5Cg0000058	+	673	75	7.62	4.75	12 (6 + 6)		44	Cyto/Nucl
	***AsMT1*_chr7C**	AVESA.00001b.r3.7Cg0001922	−	487	72	7.29	5.00	13 (6 + 7)		41	Cell mem/Cyto/Nucl
MT2	***AsMT2*_chr1C**	AVESA.00001b.r3.1Cg0000164	+	529	79	7.59	5.10	14 (8 + 6)	CC, CxC, CxxC, C	41	Cell mem/Cyto/Nucl
	*AsMT2*_chr3A	AVESA.00001b.r3.3Ag0000375	−	565	79	7.56	5.05	14 (8 + 6)		41	Cell mem/Chlo/Cyto/Nucl
	*AsMT2*_chr3C	AVESA.00001b.r3.3Cg0000320	+	531	79	7.61	4.71	14 (8 + 6)		41	Cell mem/Chlo/Cyto/Nucl
	*AsMT2*_chr1D	AVESA.00001b.r3.1Dg0002311	−	390	80	7.59	6.47	17 (8 + 9)		37	Cyto
	*AsMT2*_chr4A	AVESA.00001b.r3.4Ag0003869	−	323	75	7.19	5.57	15 (8 + 7)		38	Cyto
	*AsMT2a*_chr4D	AVESA.00001b.r3.4Dg0003379	−	339	81	7.72	4.96	17 (8 + 9)		39	Cyto
	*AsMT2b*_chr4D	AVESA.00001b.r3.4Dg0003380	−	341	81	7.70	4.96	17 (8 + 9)		39	Cyto
	*AsMT2c*_chr4D	AVESA.00001b.r3.4Dg0003934	−	334	80	7.63	4.96	17 (8 + 9)		38	Cyto
	*AsMT2*_chr7A	AVESA.00001b.r3.7Ag0000076	+	388	80	7.53	6.47	17 (8 + 9)		37	Cyto
MT3	***AsMT3*_chr3A**	AVESA.00001b.r3.3Ag0000802	−	530	64	6.81	4.85	10 (4 + 6)	C, CxC	32	Nucl
	*AsMT3*_chr3C	AVESA.00001b.r3.3Cg0000779	−	910	63	6.66	5.07	10 (4 + 6)		31	Nucl
	*AsMT3*_chr3D	AVESA.00001b.r3.3Dg0000256	−	887	63	6.68	5.07	10 (4 + 6)		31	Nucl
MT4	*AsMT4*_chr1A	chr1A:252750400..252750656	+	249	82	7.91	7.36	17 (6 + 6 + 5)		16, 15	Cyto
	*AsMT4*_chr1C	AVESA.00001b.r3.1Cg0001463	+	243	80	7.83	7.36	17 (6 + 6 + 5)		14, 15	Cyto
	***AsMT4*_chr1D**	chr1D:243956192..243956442	+	249	82	7.94	7.36	17 (6 + 6 + 5)		16, 15	Cell mem/Cyto
	*AsMT4*_chr4C	chr4C:76127001..76127600	+	360	89	8.62	5.75	17 (6 + 6 + 5)		26, 13	Cell mem/Cyto

Cyto—cytoplasm, Nucl—nucleus, Cell mem—cell membrane, Chlo—chloroplast.

## Data Availability

The datasets generated during and/or analyzed during the current study are available from the corresponding author on request.

## Supplementary Materials

The following supporting information can be downloaded at: https://www.mdpi.com/article/10.3390/antiox12101865/s1, Table S1: Cis-regulatory elements identified in *A. sativa* metallothionein genes using the PlantCARE database (http://bioinformatics.psb.ugent.be/webtools/plantcare/html/, accessed on 25 May 2022).; Table S2: The numbers of particular types of cis-regulatory motifs in each type of *A. sativa* metallothionein genes and the total number of particular types of regulatory motifs in all *AsMT* genes.
